# The American Illustrated Medical Dictionary

**Published:** 1902-01

**Authors:** 


The American Illustrated Medical Dictionary. For Practitioners and Students. A Complete Dictionary of the Terms used in Medicine, Surgery, Dentistry, Pharmacy, Chemistry, and the kindred branches, including much collateral information of an encyclopedic character, together with new and elaborate tables of Arteries, Muscles, Nerves, Veins, etc.; of Bacilli, Bacteria, Micrococci; Eponymic Tables of Diseases, Operations, Signs and Symptoms, Stains, Tests, Methods of Treatment, etc., etc. By W. A. Newman Dorland, A. M., M. D., Editor of the "American Pocket Medical Dictionary." Second edition, revised. Handsome large octavo, nearly 800 pages, bound in full flexible leather. Philadelphia and London: W. B. Saunders & Co. 1901. Price, $4.50 net.

This dictionary, while of convenient size for quick reference, contains all that is of practical value in even the largest and most complete works of its kind. Avoiding their objectionable features it retains the advantages of both the unabridged lexicon and the too much abridged pocket dictionary.

Besides the ordinary dictionary matter about one hundred and twenty-five carefully prepared tables have been added. In addition to the ordinary clinical matter so arranged there are tables on tests, staining methods, methods of treatment and the like.

A number of new terms which appeared in recent medical literature are included in the present revised edition.

Altogether the work deserves well at the hands of the profession. Considering its character, the price is merely nominal. R.

